# Comparative Bioactivity Evaluation of Chemically Characterized Essential Oils Obtained from Different Aerial Parts of *Eucalyptus gunnii* Hook. f. (Myrtaceae)

**DOI:** 10.3390/molecules28062638

**Published:** 2023-03-14

**Authors:** Hocine Abbaci, El-hafid Nabti, Abdullah M. Al-Bekairi, Soheir A. A. Hagras, Mounir M. Salem-Bekhit, Abdenour Adjaoud, Hayat Ali Alzahrani, Leila Bensidhoum, Rawaf Alenazy, Alessandra Piras, Danilo Falconieri, Silvia Porcedda, Yacine Benguerba, Karim Houali

**Affiliations:** 1 Laboratoire de Maitrise des Energies Renouvelables, Faculté des Sciences de la Nature et de la Vie, Université de Bejaia, Bejaia 06000, Algeria; 2 Inaya Medical Colleges, Riyadh 11352, Saudi Arabia; 3 Department of Clinical Laboratory Sciences, Inaya Medical Colleges, Riyadh 11352, Saudi Arabia; 4 Department of Drug Radiation Research, National Center for Radiation Research and Technology (NCRRT), Egyptian Atomic Energy Authority, Cairo 11787, Egypt; 5 Department of Pharmaceutics, College of Pharmacy, King Saud University, Riyadh 11451, Saudi Arabia; 6 Département des Sciences Biologiques de l’Environnement, Faculté des Sciences de la Nature et de la Vie, Université de Bejaia, Bejaia 06000, Algeria; 7 Department of Medical Laboratory Technology, Faculty of Applied Medical Sciences, Northern Border University, Arar 73211, Saudi Arabia; 8 Department of Medical Laboratory, College of Applied Medical Sciences-Shaqra, Shaqra University, Shaqra 11961, Saudi Arabia; 9 Department of Chemical and Geological Sciences, University of Cagliari, Cittadella Universitaria, 09042 Monserrato, Italy; 10 Technical Institute “Michele Giua”, Via Montecassino, 09134 Cagliari, Italy; 11 Laboratoire de Biopharmacie Et Pharmacotechnie (LBPT), Université Ferhat ABBAS Sétif-1, Sétif 19000, Algeria; 12 Laboratoire de Biochimie Appliquée et Biotechnologies (LABAB), Faculté des Sciences Biologiques et des Sciences Agronomiques, Université Mouloud MAMMERI de Tizi-Ouzou, Tizi-Ouzou 15000, Algeria

**Keywords:** *Eucalyptus gunnii*, chemical composition, insecticidal, antibacterial, essential oils, *Callosobruchus maculatus*, Pectobacteriaceae

## Abstract

Essential oils (EOs) obtained by hydro-distillation from different parts of twigs (EOT), leaves (EOL), and fruits (EOF) of *Eucalyptus gunnii* Hook. f. were screened for their chemical composition, insecticidal, repellence, and antibacterial properties. Based on GC and GC/MS analysis, 23 constituents were identified across the twigs, leaves, and fruits, with 23, 23, and 21 components, respectively. The primary significant class was oxygenated monoterpenes (82.2–95.5%). The main components were 1,8-cineole (65.6–86.1%), α-terpinyl acetate (2.5–7.6%), o-cymene (3.3–7.5%), and α-terpineol (3.3–3.5%). All three EOs exhibited moderate antibacterial activities. EOL was found to have higher antibacterial activity against all tested strains except *Dickeya solani* (CFBP 8199), for which EOT showed more potency. Globally, *Dickeya solani* (CFBP 8199) was the most sensitive (MIC ≤ 2 mg/mL), while the most resistant bacteria were *Dickeya dadantii* (CFBP 3855) and *Pectobacterium carotovorum* subsp. *carotovorum* (CFBP 5387). Fumigant, contact toxicity, and repellent bioassays showed different potential depending on plant extracts, particularly EOT and EOL as moderate repellents and EOT as a medium toxicant.

## 1. Introduction

*Eucalyptus* stands are a prominent feature of the Australian and Tasmanian natural landscapes [1]. The genus *Eucalyptus* L’Heritier is the largest representative of the Myrtaceae family with approximately 900 species and subspecies worldwide [2]. Most of the *Eucalyptus* species are native to Australia. They are fast-growing timber species, much faster than most European or North American trees. Consequently, *Eucalyptus* species are increasingly becoming the trees of choice in planting projects around the world for production of fuels, chemicals, and materials [3]. Outside of its native area, Tasmania, *E. gunnii* Hook. f. is in particular widely grown throughout Britain and Ireland [2]. Although we were not able to find any official report on the number of introduced *Eucalyptus* species, it is documented that *E. globulis* Labill. was the first species brought by Ramel in Algeria in 1854 [4]. The first raised micro-plantations in Algeria were not primarily intended to generate revenue but for draining excess water in frequently waterlogged areas to prevent malaria. *Eucalyptus* has a long history in the country, first planted to preserve public health by reducing disease transmission as malaria ravaged the Algerian population between 1867 and 1876. Presently, Eucalypti plantations occupy relatively small areas in the country where *E. gunnii* seems to be by far a less favored species. Indeed, other types of *Eucalyptus* such as *E. globulis* Labill. and *E. camaldulensis* Dehn. are the most commonly grown. This is probably why, locally, these two species have been studied intensively, but little attention has been paid to less frequently planted species such as *E. gunnii.* Cider gum’s main center of distribution is Tasmania, to which it is endemic [5]. Aboriginal people extract their drinks, known as *way-a-linah*, from the sap of the tree. These alcoholic beverages have existed for thousands of years in Tasmania [6]. Cider gum and other eucalypts have been used traditionally worldwide not only as a source of food or beverage but also to treat many external or internal complaints, such as colds, fevers, and diarrhea. Indeed, due to their various attributes, many *Eucalyptus* species have been listed in the pharmacopeias of many countries [7]. This large genus of around 900 species and subspecies has proven to possess a considerable number of health-promoting bioactive compounds. *Eucalyptus* spp. are well known for their high contents in useful volatiles, particularly 1,8-cineole, commonly used in the medicinal and perfumery industries. Several *Eucalyptus* spp. contain more than 70% content of 1,8-cineole [8,9,10], including our investigated plant. *E. gunnii* as a potential source of biomass and bioenergy [11,12] raised interest in the qualitative improvement of plant regeneration, in particular genetic engineering. In contrast, very few studies have been published in relation to the chemical composition [13,14,15] and bioactivity of cider gum [14,15,16,17,18]. Little information is available on the bioactive compounds of EOs extracted from E. *gunnii* worldwide. Moreover, no part of this plant has been investigated before in Algeria, probably due to its limited distribution. *E. gunnii* twigs, leaves, and fruits were harvested from one of the rarest known plantations (Northern Algeria), and the phytocomponents of the different extracted EOs were analyzed and identified using combined GC and GC/MS. For better knowledge of the plant potential, the obtained three EOs were tested separately under the same conditions for their antimicrobial efficacy against three different Gram-negative strains *Dickeya dadantii*, *Dickeya solani*, and *Pectobacterium carotovorum* subsp. *carotovorum*. These two genera belonging to Pectobacteriaceae [19] are widespread globally and have a broad host range of vegetable crops, including potatoes, cabbages, lettuces, and onions. These pathogens cause important economic damage [20]. Few chemical bactericides are available for their management, with variable degrees of success [21,22]. Cowpea *Vigna unguiculata* (L.) Walp. stands out in the country for its considerable contribution as a source of proteins and energy for middle-income inhabitants. Due to damage to cowpea grains caused by *Callosobruchus maculatus* (F.), various integrated pest management (IPM) approaches have emerged to control this pest, including research towards substitution of hazardous synthetic insecticides, namely the use of EOs as eco-friendly natural fumigants and contact insecticides. In this context, the study also aimed to test the bioactivity of our EOs against this stored product pest.

## 2. Results and Discussion

### 2.1. Essential Oil Yields

*E. gunnii* leaves exhibited the highest EO yield (3%), followed by fruits (0.4%) and then twigs (0.2%). Hydro-distillation of the *E. gunnii* leaves yielded a higher rate than those reported by previous studies on the same species. Indeed, the study of [17] on 13 sampled *Eucalyptus* species from Tunisia reported the lowest EO yield for *E. gunnii* (0.5%) and the highest rate for *Eucalyptus cinerea* (3.9%). EO of *E. gunnii* leaves from Southern Italy yielded 1.1% [15]. Our results do not match those obtained by [17]. Still, they agree with [14], where investigated Australian samples yielded a higher amount of EO (2.1%) than those of Southern Italy (1.1%). These variations in EO yields were probably due to different environmental factors. The age and sampling season may also affect the EO yield. EO yields, particularly 1,8-cineole, were found to be dependent on the *Eucalyptus* source, and several studies revealed variability in EO content in the same species [7]. Our plant material was harvested at the entire flowering stage from a relatively young plantation. A study by [23] confirmed these significant differences in EO yields for juvenile and adult leaves among several *Eucalyptus* species, including *E. gunnii.*

### 2.2. Chemical Composition

Data obtained by GC and GC/MS analysis of *E. gunnii* EOs have been compiled in Table 1. The results revealed noticeable quantitative variation in the EOs for both major and minor compounds. Despite the chemical composition in all EOs showing qualitative similarities, it is interesting to note that most of the compounds for EOL (11 out 21) and EOF (15 out 21) were detected as traces. The total number of identified compounds in all EOs was 23, distributed as 23, 23, and 21 compounds for twigs, leaves, and fruits, respectively. In [18], almost the same number of compounds (24 in total) was identified, accounting for 97.9% of the total EO. However, 30 compounds were identified for EO of *E. gunnii* leaves from Southern Italy [15]. For our tree, the major EOF constituents were 1,8-cineole (86.1%), α-terpinyl acetate (4.2%), α-terpineol (3.5%), and ortho-cymene (3.3%). The main EOL constituents were 1,8-cineole (83.3%), α-terpineol (4.6%), and ortho-cymene (3.3%), while significant compounds found in EOT were 1,8-cineole (65.6%), α-terpinyl acetate (7.6%), ortho-cymene (7.5%), and α-terpineol (3.6%). EOs of *Eucalyptus* species are well known to be rich in 1,8-cineole [7,24,25]. Indeed, for our EOs, the oxygenated monoterpenes were the first significant class (95.5, 93.7, and 82.2% in fruits, leaves, and twigs, respectively) represented essentially by 1,8-cineole. 1,8-Cineole was found to be highly abundant (up to 86% in fruits) compared to all the previous studies on *E. gunnii* EOs. Ref. [15] reported that oxygenated monoterpenes (80.3%) were the dominant class of *E. gunnii* EO extracted from leaves from Southern Italy. According to Table 1, monoterpene hydrocarbons (MH), which were distinguished as a second dominant class, occupied less than 12% of EO volume, essentially represented by ortho-cymene (7.5, 3.3, and 3.3% in twigs, fruits, and needles, respectively) and α-pinene ranging from 0.9%, for leaves to 2.9% for twigs. The chemical composition of our *E. gunnii* EOs is similar to what has been described in most earlier investigations, where 1,8-cineole was observed as the predominant component, with proportions ranging from 17.9 to 74.6% [14,15,18]. The exception was made for the study of [17], where 1,8-cineole (2.6%) was reported as a minor constituent. Spathulenol (16.5%) was the predominant component of *E. gunnii* EO from Tunisia [17]. Moreover, spathulenol was found in *E. gunnii* EO from Argentina, with a proportion of 12.3% [26]. This compound was absent in our sample and found in a meagre amount (0.6%) in the study of [18]. In Tunisian EO, viridiflorol (11.5%) and globulol (12.5%) were found in noticeable concentrations, while these components were present in our EO with a lower amount of less than 2%. Ref. [23] concluded that EOs of *Eucalyptus* species are either rich in 1,8-cineole and α -pinene or in p-cymene and/or spathulenol and globulol. Our EOs fall into the first category. Moreover, the same author reported that species belonging to the *Symphyomyrtus* subgenus characteristically have a high amount of 1,8-cineole and α-pinene. In comparison to the very few studies on *E. gunnii*, our results have some similarities with the data of [15] (1,8-cineole 74.7%; α-pinene 13.1%; Terpineol 4.2%) and [14] (1,8-cineole 67.80%; α-pinene 14.12%; β-phellandrene 3.92%; α-terpinyl acetate 3.27%; and α-terpineol 2.08%) but did not match the analyzed EO obtained by [17], which is much richer in spathulenol, viridiflorol, and globulol. All these results indicate that our *E. gunnii* EOs are closer to those sampled from Southern Montenegro, Tasmania, and Southern Italy but different from those analyzed in Tunisia. A significant amount of 1,8-cineole in *E. gunnii* has already been reported but not at such a high rate. Because of this very high amount of 1,8-cineole in our samples (up to 86% in fruits), we found our EOs to be richer in eucalyptol than several studied *Eucalyptus* species. It is essential to highlight that this high amount was found mainly in fruits, organs rarely involved in studies. This high rate of 1,8-cineole in our EO was remarkable, indicating the importance of our investigated tree, which could be used as an essential source of 1,8-cineole.

### 2.3. Antibacterial Activity

The results of agar diffusion assays showed antibacterial activity with variable degrees of all EOs against the tested bacteria: *D. dadantii*, *D. solani*, and *P. carotovorum* subsp. *carotovorum*. The antibacterial effect increased with the number of applied EO concentrations. EOL displayed a higher impact than EOT and EOF against all bacterial strains except for *D. solani*, where EOT exhibited the most significant inhibitory activity. Indeed, the IZD is the highest (27, 85 mm) compared to leaves and fruits EOs (Table 2). Notably, the antibacterial effect of the positive control ampicillin against tested strains was more potent than that of EOs obtained from our plant. Table 3 shows that the most susceptible strain was *D. solani* (MIC ≤ 2 mg/mL). The most resistant bacteria were *D. dadantii* and *P. carotovorum* subsp. *carotovorum*, as MICs were not observed with the highest tested concentration of 2 mg/mL. ANOVA results revealed significant differences between tested microorganisms and EOT, EOL, and EOF (*p* ≤ 0.05).

Different antimicrobial patterns for our EOs were observed. Notably, the antibacterial effect of ampicillin against all tested strains was stronger than that of EOs obtained from our plant. Among all EOs analyzed in this study, EOF was less effective. Notably, the harvesting period and extracting procedure were the same for all plant parts. This lower acceptable potency of EOF against most strains might be related to the absence of cis-pinocarveol and/or α-thujene compounds. Indeed, α-thujene is known to display antibacterial activities [27]. 1,8-Cineole was present at significant amounts in the three EOs by reaching its maximum in EOF (86.1%). Ref. [28] hypothesized that eucalyptol is a main player in antimicrobial activity. However, other researchers [28,29] reported that 1,8-cineole might be responsible in some cases for low antimicrobial activities. Phytochemically speaking, EOF had the lowest activity, where most of the compounds (15 out 21) were detected as traces, namely α-pinene, β-phellandrene, α-terpinene, endo-fenchol, trans-pinocarveol, pinocarvone, borneol, cis-carveol, (Z)-ocimenone, thymol, carvacrol, (Z)-jasmone, globulol, viridiflorol, and rosifoliol. Nevertheless, the lower inhibitory activity of EOF might not be due to these components present in traces. There are some reports that confirm some Lamiaceae EOs’ inhibition against some Pectobacteriaceae. For example, [30] showed the significant antibacterial properties of *Lavandula angustifolia* and *Rosmarinus officinalis* against *P. carotovorum* subsp. *carotovorum* and moderate effect against *D. solani* and *P. carotovorum* subsp. *atrosepticum*. It should be noted that, globally, IZD values of EOL and EOT are similar, a basis for considering both as potential natural agents. However, this potential cannot be observed in all eucalypts. For example [31], reported the weak antibacterial activity of *E. glubulus* EO in controlling *P. carotovorum* subsp. *carotovorum*. Globally, many authors detected weaker activity of the EOs against *D. solani* and *D. dadantii.* Some tested plant extracts revealing weak activity were from Lamiaceae: *Lavandula angustifolia*, *L. latifolia*, *Melissa officinalis*, *Mentha pulegium*, *Origanum majorana*, and *Thymus mastichina* [32]; *T. vulgaris*; and *Rosmarinus officinalis* [31]. Ref. [18] reported good antibacterial activity, with a MIC value of 2 µg/mL for *P. carotovorum.* Our results on *P. carotovorum* subsp. *carotovorum* disagreed with those reported by [18]. This could be due to the nature of the experimental design or the fact that the tested *P. carotovorum* was less resistant than our reference strain. Notably, the subspecies of *P. carotovorum* were not precisely indicated by the authors. The inhibitory potential may differ for the same bacteria according to the nature of in vitro or in vivo tests [33]. Considering these relatively inconsistent inhibitory effects, consideration should be given to the phytochemical variability in all EOs (qualitatively and quantitatively). Notably, our GC and GC/MS analysis revealed 1,8-cineole to be the major constituent for all our EOs. Ref. [28] suggested that the activity of eucalyptol towards specific pathogens depends on its percentage share in individual plants. However, it is not easy to attribute the exhibited antibacterial activity to this compound. From the outcome of our investigation, it is possible to conclude the potential activities of *E. gunnii* EOs against many Pectobacteriaceae, even though results exhibited moderately distinct patterns of potency against the tested strains.

### 2.4. Insecticidal and Repellent Activities

Insecticidal and repellent activities were investigated against *C. maculatus* using all extracted EOs (twigs, leaves, and fruits) obtained from *E. gunnii*. Obtained data for the contact toxicity test (Table 4) revealed that EOL caused the highest mortality to *C. maculatus*, followed by EOT and EOF with LD_50_ values of 2.153, 5.933, and 5.986 µL/mL, respectively. For LD_95_, EOL (12,647.893 µL/mL) and EOF (1958.130 µL/mL) evoked practically no significant mortality dose effect on *C. maculatus*. For LD_95_, EOT was the most effective, with a 153.825 µL/mL value. For LT_50_, EOF (LT_50_= 15.282 h) could kill 50% of individuals more rapidly than EOL and EOT.

After 4 days, post-exposure results for the fumigant toxicity test (Table 5) revealed that EOT achieved the highest insecticidal activity against the stored product pest *C. maculatus* with LD_50_ values of 1.788 µL/mL followed by EOF (LD_50_ = 4.089 µL/mL) and EOL (LD_50_ = 4.444 µL/mL). For LD_95_, EOT achieved the highest toxicity with a 45.643 µL/mL value. Globally, EOT was the most toxic oil, followed by EOL and EOF. For LT_50_, EOT (LT_50_= 64.197) was more effective in causing the death of 50% of individuals than EOL and EOF.

The results of the evaluation of the repellent effects on *C. maculatus* have shown that EOT at concentrations of 2, 4, 8, and 16 µL/mL caused repellency of 30 ± 25.81, 50 ± 11.55, 60 ± 16.33, and 70 ± 11.55%, respectively. EOT recorded the highest mean repulsion rate of 52.5 ± 6.73%, falling into class III (good repellent), calculated according to [34]. Similar results were observed for EOL, which share the same class. Smaller effects were observed in EOF, considered a moderate repellent (class II) (Table 6).

For each bioassay, all EOs were tested under the same experimental conditions against *C. maculatus*, a serious insect pest causing potential damage to economically important *Vigna* crops such as *V. unguiculata* (cowpea), *V. mungo* (black gram), *V. angularis* (azuki bean), and *V. radiata* (mungbean) [35]. EOs obtained from twigs, leaves, and fruits demonstrated repellence effect, contact, and fumigant toxicity on *C. maculatus*. For each test, there were different responses in EO efficacy at the tested doses, as shown in Table 4, Table 5 and Table 6. The results indicated that the insecticidal activity of the EOs varied depending on the nature of the bioassay and the used plant part. The efficacy in respect of the contact toxicity for LD_50_ was in the order leaves > twigs > fruits. For LD_95_, the order was twigs > fruits> leaves, and for LT_50_ it was fruits > leaves > twigs (Table 4). Responses within 96 h post-treatment for the fumigant toxicity were in the order twigs > fruits > leaves. For LD_95_ the order was twigs > leaves > fruits, and for LT_50_ it was twigs > leaves > fruits (Table 5). These differences in the precedence of the most toxic EOs within contact and fumigant toxicity tests might be due to differences in the active chemical compounds of each EO that operate via several modes of action within each test. For the fumigant test, the toxic properties of EOT were more efficient than EOL and EOF within 96 h post-treatment. For LT_50_, EOT’s rapid action against the bruchid beetle might be due to the accessibility of specific active chemical groups of the tested EO causing rapid suffocation and then lethal effect. The repellence of EOs on *C. maculatus* has shown relatively moderate to good results depending on concentrations and plant extracts (Table 6). According to the classification established by [34], it can be concluded that EOT recorded a good repellent effect (class III) against *C. maculatus* adults, slightly ahead of EOT. However, EOF showed lower repellent effects (class II) compared to EOT and EOL in our study, illustrating that fruit extract was the less toxic EO. In the literature, several studies using different bioassays and protocols (inhalation, fumigation, contact, repulsion, etc.) have been conducted on diverse EOs or commercially available EO-based products against *C. maculatus*. Still, no prior reports are available on the activity of *E. gunni* EOs against this bruchid beetle. A unique insecticidal activity study of *E. gunni* was the evaluation of its EO on larvicidal effect in *Aedes aegypti* (L.) (Diptera: Culicidae) by [26]. The author observed that increasing the content of 1,8-cineole reduced the larvicidal effect in *Ae. aegypti*. Conversely, most reports have shown various species of Eucalyptus exhibiting moderate to highly toxic activity against several major stored-product insects, including bruchid beetles such as *C. maculatus*. The insecticidal activity of many plants’ EOs might be attributed to their monoterpenoid contents [36,37]. Indeed, this large class was reported by many authors as fumigants and contact toxicants in several major stored-product insects. Specifically, many researchers demonstrated that EOs with high contents in 1,8-cineole [38,39], terpineol, and α-pinene [39] induced high repellent and toxic effects against several major stored-product insects. For our EOs, the oxygenated monoterpenes were the first major class, representing 95.5, 93.7, and 82.2% in fruits, leaves, and twigs, respectively. The results obtained for fumigant and contact toxicity tests revealed that all tested EOs were moderately bioactive towards *C. maculatus*. Moreover, EOT had the lowest content of all plant parts in 1,8-cineole (65.6%) but was the most effective EO as a repellent and toxicant. Globally, fumigant, contact toxicity, and repellent bioassays showed different potential depending on plant extracts, particularly EOT and EOL as good repellents and EOL as a moderate toxicant. Ref. [40] demonstrated that a high amount in 1,8-cineole and α-terpinyl acetate, which took up about 70% of *Laurus nobilis* and was similar to our EOs, did not seem to greatly affect the red flour beetle *Tribolium castaneum* even at the maximum testing concentration. From the above-cited studies on the insecticidal properties and the importance of monoterpenoids and phenolic acids as toxicants, it can be concluded that common chemicals found in our EOs with a predominance of oxygenated monoterpenes (82.2–95.5%) (1,8-cineole (65.6–86.1%), α-terpinyl acetate (2.5–7.6%), ortho-cymene (3.3–7.5%), and α-terpineol (3.3–3.5%)) might not be directly responsible for the moderate activity of the EOs. These major compounds could not completely determine the overall effect of our EOs. Specifically, antagonistic effects of combinations of major and/or trace compounds may occur. The evaluation of lethal concentrations (LD_50_ and LD_95_) was of particular interest. The time to achieve a lethal effect (LT_50_) led to the conclusions that (i) some common chemicals found in our EOs, such as 1,8-cineole, might be responsible for low activity; (ii) higher concentrations of EOs and longer exposure time were required to achieve high repellence and toxicity against *C. maculatus*; and (iii) higher susceptibility of *C. maculatus* to EOs was observed in fumigation test than in the contact toxicity bioassay.

## 3. Material and Methods

### 3.1. Plant Material and Oils Extraction

Healthy aerial parts of *E*. *gunnii* Hook. f. (Myrtaceae) were harvested at the complete flowering stage (June 2020) from a small, planted population located in the Béjaia region, Northeastern Algeria (latitude 36°362′11.262″2‴ (N); longitude 10°302′1.902″2‴ (E); altitude 200 m). The plant material was shade-dried at an ambient temperature of 25–28 °C for several days until reaching a constant weight before EO extraction and the outcoming assays. Leaves, twigs, and fruits were weighed separately, and 100 g for each air-dried aerial part was subject to hydro-distillation for 3 h using a Clevenger-type apparatus. The different EOs were dehydrated using anhydrous sodium sulfate and stored in dark glass vials at 4 °C until needed.

### 3.2. Chemical Analysis

The EOs were analyzed by gas chromatography (GC) and gas chromatography–mass spectrometry (GC–MS). The samples were examined by GC Agilent apparatus (Model 6890N, Palo Alto, CA, USA) equipped with an autosampler (model 7683), a split/splitless injector, and a capillary chromatographic column HP-5 (5% phenyl-methylpolysiloxane, 30 m; 0.25 mm; 0.25 μm) programmed in the temperature range 60–240 (3 °C/min) followed by 20 min under isothermal conditions. The injector was maintained at 250 °C. The carrier gas type was helium, and a 1 μL sample was injected in the split mode (1:10) at a rate of 1.0 mL/min. The GC was fitted with a quadrupole mass spectrometer, MS, Agilent model 5973 detector. MS was performed under the following conditions: ionization energy 70 eV, electronic impact ion source heating at 200 °C, quadrupole temperature fixed at 150 °C, scan rate 3.2 scans/s, mass range 30–480 u. Chemstation software was used to handle mass spectra and chromatograms and measure peak areas. The percentage of individual components was calculated based on GC peak areas without FID response factor correction. Samples were run in hexane with a dilution ratio of 1:100. Identification of the chemical components was based on a comparison of inhouse library where the retention indexes (RIs) were defined based on the n-alkane scale (2 standard mixes C8–C20 and C21–C40). The nature of components was also determined by other mass spectral data gathered by the Adams library [41], various MS libraries, and reported data in the literature by comparing compounds’ elution order and retention indexes on semi-polar phases.

### 3.3. Bacterial Cultures

Three Gram-negative strains were used for evaluating the antibacterial activity: *Dickeya dadantii* (CFBP 3855), *Dickeya solani* (CFBP 8199), and *Pectobacterium carotovorum* subsp. *carotovorum* (CFBP 5387). Dr. Ladjouzi R. (University of Béjaia, Algeria) kindly provided all strains. The cultures were maintained by periodic subcultures in nutrient broth, and the bacterial strains were preserved at −80 °C for further investigation.

### 3.4. Antibacterial Screening

To determine the antibacterial activity of our EOs, we opted for the agar diffusion assay, a well-established procedure considered one of the most commonly used antimicrobial susceptibility testing methods [42] approved by the Clinical and Laboratory Standards Institute [43]. After a McFarland 0.5 turbidity standard was obtained, sterile cotton swabs were used to spread our bacterial suspensions on the surface of Muller–Hinton Agar plates. A set of three sterile filter paper disks (Whatman no. 1, 6 mm diameter) were placed on the surface of the media of each Petri dish (9 cm in diameter). Filter paper disks were impregnated with 10 μL of each dilution of *E. gunnii* EOs (*v*/*v*; 1/2, 1/5, and 1/10 in DMSO). The plates were left for 30 min at room temperature to allow better diffusion before incubation. After 24 h of incubation at 36 ± 1, the inhibition zone diameter (IZD) was evaluated. Each experiment was performed in triplicate and repeated twice for all evaluated microorganisms. Sterile discs with 10 μL pure DMSO and ampicillin (10 μg/disk) were used as negative and positive controls, respectively.

The minimal inhibitory concentration (MIC) of our EOs was evaluated using the microdilution broth method performed in sterile 96-well microplates filled with 95 µL of culture medium and 5 µL of bacterial suspension using a 0.5 McFarland turbidity standard, all mixed with 100 µL of EO at different concentrations. The plates were incubated at 36 ± 1 °C for 24 h [44]. All bacterial strains were tested for possible MIC values ranging from 0.0156 to 2 mg/mL. More precisely, we tested the following EO concentrations: 0.0156, 0.0312, 0.0625, 0.125, 0.25, 0.5, 1.0, and 2.0 mg/mL.

### 3.5. Insect Rearing and Sampling

The stock colonies of *Callosobruchus maculatus* (F.) used in this study were obtained from the entomology laboratory of Mouloud Mameri University (Tizi Ouzou, Algeria). All the collected insects were placed into 2 L glass jars and reared on cleaned cowpea variety *Vigna unguiculata* (L.) Walp. Insects were maintained in a microclimate chamber with neon white light at 30 ± 1 °C, 75% relative humidity, and a 12 h photoperiod. Unsexed adult beetles (7–14 days old) were used for all experiments (contact toxicity, repellence, and fumigant assays) carried out under the same environmental conditions.

### 3.6. Contact Toxicity Bioassay

Contact toxicity of EOs from *E. gunni* aerial parts was evaluated against *C. maculatus* according to the method described by [45]. Each Petri dish (9 cm in diameter) containing 40 g of sterilized cowpea seeds received 1 mL of test solution containing the appropriate concentration of EO diluted in acetone: 2, 4, 8, 16, and 0 (control) µL/mL. After acetone was allowed to evaporate, groups of 20 non-sexed adult insects (1 to 7 days old) were placed in each treated Petri dish, maintained in an incubator, in dark conditions at 30 ± 1 °C and 75% relative humidity. Four replicates were set up for each EO concentration and control. Insect mortality was evaluated on a daily basis for 4 days. Obtained data were corrected using Abbott’s formula [46].

### 3.7. Fumigant Toxicity Bioassay

To assess the fumigant toxicity of EOs on the adults of *C. maculatus* (F.), a small cotton ball attached to the lower side of a 500 mL glass jar’s lid was impregnated with a chosen EO concentration, and this was replicated four times. The control groups consisted of a similar setup with no presence of EO. The concentrations of EOs used were 2, 4, 8, 16, and 0 (control) μL/mL. The vials were screwed tightly, each containing 10 unsexed adults (7–14 days old). The glass jars were kept at 30 ± 1 °C and 75% relative humidity. Mortality counts were carried out at 24, 48, 72, and 96 h after initial exposure [47].

### 3.8. Repellence Bioassay on Filter Paper

Repellent activity against *C. maculatus* was tested using the area preference test described by [34]. The Petri dish was covered with filter paper (Whatman no. 1, 11 cm diameter) and divided into two halves: one half-disc was treated with 0.5 mL of EO solutions in acetone at doses 2, 4, 8, and 16 μL/mL. In contrast, the other half was not treated and used as a negative control (acetone only). After acetone was allowed to evaporate completely from the treated and untreated paper semi-discs, 20 unsexed adults were placed in the center of each Petri dish and then sealed with parafilm. Four replications were carried out for each tested concentration. After one hour, insects on both half-filters were counted separately: weevils found on the treated semi-discs were considered not repelled, while those found in the control area were regarded as repelled. Percentage repellence (PR) values were calculated as follows: PR% = (NC − NT/NC + NT) × 100, where NC is the number of insects present in the untreated half-disc (negative control with 0.5 mL of acetone), and NT is the count obtained from the opposite treated half (with 0.5 mL of EO solutions in acetone).

As ranked by [34], the mean repellence value was calculated and assigned to one of the different repellent classes ranging from 0 to V: class 0 (PR= 0.1%), class I (PR = 0.1–20%), class II (PR = 20.1–40%), class III (PR = 40.1–60%), class IV (PR = 60.1–80%), and class V (PR = 80.1–100%).

### 3.9. Data Analysis

The relative percentage values in the contact and fumigant toxicity tests were adjusted for insecticidal activity using Abbott’s formula: M (%) = (MT−MC/100−MC) × 100, where M is the corrected insect mortality, MT is the insect mortality in the treated population of insect while MC is the insect mortality observed in the control. Data obtained from dose–response for contact and fumigation tests were analyzed with the probit method using US EPA program version 1.5 [48] to calculate LD_50_, LD_95,_ and LT_50_ values of the EOs. We conducted a two-way analysis of variance for antibacterial activity. The test was considered statistically significant at *p* < 0.05. Statistical results were obtained using the software XLSTATS (version 2022).

## 4. Conclusions

As far as the literature is concerned, this study represents the first Algerian report on *Eucalyptus gunnii* targeting phytochemical analysis and antibacterial and insecticidal effects of three different extracted EOs (twigs, leaves, and fruits). The GC and GC/MS analysis indicated the presence of 23 different chemicals, with 1,8-cineole, α-terpinyl acetate, o-cymene, and α-terpineol being the main constituents. In all EOs, 1,8-cineole was identified as the major oil component, remarkably (65.6–86.1%) higher than most previously studied *Eucalyptus* species, making the tree a valuable source of eucalyptol. Moderate antibacterial activity was observed. Specifically, EOL was found to have higher antimicrobial activity against all tested strains except *Dickeya solani*, for which EOT was more potent. Overall results of the fumigant, contact toxicity, and repellent bioassays on *C. maculatus* showed variable potential depending on plant extracts, particularly EOT and EOL as effective repellents and EOT as a moderate toxicant.

## Figures and Tables

**Table 1:** Compositions of the essential oils obtained from twigs, leaves, and fruits of *Eucalyptus. gunnii* collected in Northern Algeria.

No.	RI	RT	Compound	Twigs (%)	Leaves (%)	Fruits (%)
Monoterpene hydrocarbons (MH)				11.3	4.3	4.4
1	929	5.0423	α-thujene	0.1	0.1	-
2	937	5.2172	α-pinene	2.9	0.9	1.1
3	979	6.3057	β-pinene	0.1	Tr	Tr
4	1006	7.0926	α-phellandrene	0.2	Tr	Tr
5	1031	7.8445	ortho-cymene	7.5	3.3	3.3
6	1061	8.8587	γ-terpinene	0.5	Tr	Tr
Oxygenated monoterpenes (OM)				82.2	93.7	95.5
7	1040	8.1243	1,8-cineole	65.6	83.3	86.1
8	1113	10.8085	endo-fenchol	0.1	0.1	Tr
9	1140	11.7921	trans-pinocarveol	1.3	0.8	Tr
10	1163	12.7188	pinocarvone	0.5	0.2	Tr
11	1166	12.8412	borneol	0.1	Tr	Tr
12	1178	13.3309	terpinen-4-ol	1.1	1.5	1.7
13	1188	13.7636	cis-pinocarveol	0.8	0.7	-
14	1191	13.921	α-terpineol	3.6	4.6	3.5
15	1228	15.3899	cis-carveol	0.4	Tr	Tr
16	1231	15.521	(Z)-ocimenone	0.3	Tr	Tr
17	1287	17.8948	thymol	0.3	Tr	Tr
18	1301	18.533	carvacrol	0.3	Tr	Tr
19	1351	20.5439	α-terpinyl acetate	7.6	2.5	4.2
20	1396	22.5024	(Z)-jasmone	0.2	Tr	Tr
Sesquiterpene hydrocarbons (SH)				2.5	0.6	Tr
21	1581	29.7636	globulol	2.0	0.6	Tr
22	1588	30.0478	viridiflorol	0.3	Tr	Tr
23	1598	30.4587	rosifoliol	0.2	Tr	Tr
Non-identified (other compounds)				3.5	1.1	Tr
Identified compounds				96	98	99.9

RT: retention time. RI: retention index. Tr: trace. -: not detected.

**Table 2:** Antibacterial activities of *Eucalyptus gunnii* essential oils and ampicillin as reference antibiotics.

		Inhibition Zone Diameter (IZD) (mm) *									
	**Plant Parts**	**Twigs EO**			**Leaves EO**			**Fruits EO**			Ampicillin **
	*Essential oils Concentrations*	1/2 (V/V)	1/5 (V/V)	1/10 (V/V)	1/2 (V/V)	1/5 (V/V)	1/10 (V/V)	1/2 (V/V)	1/5 (V/V)	1/10 (V/V)	10 μg/disc
*Bacterial strains*	*Dickeya dadantii* CFBP 3855	11.69 ± 1.35 ^efgh^	11.52 ± 1.13 ^efghi^	9.14 ± 1.22 ^hij^	19.84 ± 1.7 ^c^	12.21 ± 1.27 ^efg^	8.72 ± 1.56 ^ij^	10.26 ± 0.87 ^fghij^	8.67 ± 1.37 ^ij^	8.01 ± 1.12 ^j^	31.15 ± 1.5
	*Dickeya solani* CFBP 8199	27.85 ± 1.97 ^a^	13.31 ± 1.64 ^e^	11.8 ± 0.40 ^efgh^	22.30 ± 1.67 ^b^	12.88 ± 1.85 ^ef^	10.40 ± 1.49 ^fghij^	16.97 ± 2.07 ^d^	11.80 ± 1.19 ^efghi^	9.23 ± 1.33 ^ghij^	30.79 ± 1.3
	*Pectobacterium carotovorum* subsp. *carotovorum* CFBP 5387	10.98 ± 1.43 ^efghij^	9.9 ± 1.59 ^ghij^	9.15 ± 0.58 ^hij^	17.85 ± 2.87 ^d^	10.01 ± 2.05 ^ghij^	8.26± 1.20 ^j^	8.53 ± 1.80 ^j^	8.35 ± 1.27 ^j^	8.52 ± 1.5 ^j^	33.06 ± 1.9

* Inhibition zone diameter (IZD): values include the diameter of the disc (6 mm). ** Ampicillin used as a positive control. Values were expressed as mean where all experiments were repeated twice in triplicate. Mean values in columns followed by different superscript letters show statistical difference according to the Student–Neuman–Keuls test (*p* < 0.05).

**Table 3:** The minimum inhibitory concentration of *Eucalyptus gunnii* essential oils against the tested bacterial strains.

Bacterial Strain		MIC (mg/mL)
Fruits EO	*Dickeya dadantii* CFBP 3855	>2
	*Dickeya solani* CFBP 8199	≤2
	*Pectobacterium carotovorum* subsp. *carotovorum* CFBP 5387	>2
Twigs EO	*Dickeya dadantii* CFBP 3855	>2
	*Dickeya solani* CFBP 8199	≤2
	*Pectobacterium carotovorum* subsp. *carotovorum* CFBP 5387	>2
Leaves EO	*Dickeya dadantii* CFBP 3855	>2
	*Dickeya solani* CFBP 8199	>2
	*Pectobacterium carotovorum* subsp. *carotovorum* CFBP 5387	>2

**Table 4:** The results of probit analysis for contact toxicity of *Eucalyptus gunnii* essential oils against *Callosobruchus maculatus* after 96 h exposure time.

Plant Parts	LD_50_ (µL/mL) (Min–Max)	LD_95_ (µL/mL) (Min–Max)	Fit of Probit Line				LT_50_ (Hours) (Min–Max)
			*χ^2^*	*p*. Value	Intercept	Slope ± SE	
Leaves	2.153 (0.000–4.580)	12,647.893 (284.470–α)	0.134	0.05	4.854	0.436 ± 0.21	85.735 (78.244–95.947)
Twigs	5.933 (4.421–8.069)	153.825 (61.455–1075.994)	3.431	0.05	4.100	1.163 ± 0.218	117.401 (101.475–143.982)
Fruits	5.986 (3.298–11.685)	1958.130 (196.815–48,071.760)	0.046	0.05	4.491	0.654 ± 0.211	15.282 (12.755–19.377)

LD_50_: dose necessary to cause the death of 50% of the tested insects (median lethal dose). LD_95_: Sufficient dose to cause the death of 95% of the tested insects. LT_50_: average lethal time (50% survival time of the tested insects).

**Table 5:** Fumigant toxicity of three essential oils (leaves, twigs, and fruits) from *Eucalyptus gunnii* on adult *Callosobruchus maculatus* after 96 h.

Plant Parts	LD_50_ (µL/mL) (Min–Max)	LD_95_ (µL/mL) (Min–Max)	Fit of Probit Line				LT_50_ (Hours) (Min–Max)
			*χ^2^*	*p*. Value	Intercept	Slope ± SE	
Leaves	4.444 (2.818–6.275)	242.906 (73.329–5203.156)	0.204	0.05	4.387	0.946 ± 0.215	90.562 (81.630–103.375)
Twigs	1.788 (0.840–2.631)	45.643 (23.321–198.635)	0.698	0.05	4.705	1.169 ± 0.233	64.197 (59.912–69.142)
Fruits	4.089 (2.206–6.082)	427.538 (94.620–43,364.758)	2.066	0.05	4.501	0.814 ± 0.213	93.971 (85.011–106.729)

LD_50_: dose necessary to cause the death of 50% of the tested insects (median lethal dose); LD_95_: Sufficient dose to cause the death of 95% of the tested insects. LT_50_: average lethal time (50% survival time of the tested insects). Lower and upper confidence limits are shown in parentheses.

**Table 6:** Repulsion means percentages of *Eucalyptus gunnii* essential oils against *Callosobruchus maculatus* adults classified according to [34].

Plant Parts	Concentration (µL/mL)				McDonald Class (Mean ± SD)
	2	4	8	16	
Leaves	35 ± 19.15	45 ± 19.15	57.5 ± 17.07	65 ± 10	III (50.62 ± 4.34)
Twigs	30 ± 25.81	50 ± 11.55	60 ± 16.33	70 ± 11.55	III (52.5 ± 6.73)
Fruits	25 ± 10	30 ± 11.55	35 ± 19.15	40 ± 0	II (32.5 ± 7.87)

For concentration columns, each datum represents repulsion mean percentage ± SD. For each concentration, four replicates were used, each set up with 20 individuals (n = 80). For the McDonald class column, the mean repellence values ± SD were evaluated and assigned to one of the different repellent classes ranging from 0 to V as ranked by [34].

## Data Availability

Not applicable.

## Sample Availability

Samples of the compounds are not available from the authors.
